# Description of the Clinical Findings Associated With the Epizootic Hemorrhagic Disease in Cattle From Northwestern Spain During the Emergence

**DOI:** 10.1155/tbed/7808243

**Published:** 2025-05-24

**Authors:** José Manuel Díaz-Cao, Gonzalo López-Lorenzo, Cynthia López-Novo, Pablo Díaz, Susana Remesar, Ceferino López, Patrocinio Morrondo, Gonzalo Fernández, Alberto Prieto

**Affiliations:** Department of Animal Pathology (INVESAGA Group), Faculty of Veterinary Sciences, Universidade de Santiago de Compostela, Campus Terra, Lugo 27002, Spain

**Keywords:** clinical signs, EHD, oral lesions, ruminants, vector-borne

## Abstract

Epizootic hemorrhagic disease (EHD) is an emerging disease in Europe since it was first introduced in 2022 in Italy, Spain, and Portugal. The disease is traditionally considered less severe in cattle, but outbreaks have appeared with increasing frequency in the last decades. The outbreak in Spain coursed with numerous cattle farms reporting clinical disease. Our aim in this study was to evaluate the incidence of clinical disease in cattle farms from NW Spain and describe the clinical signs and their frequency. We investigated 29 cattle farms that reported EHD and estimated an incidence of farms with clinical signs of 6.2% (*n*=467) in the study area (range: 2.3%–33.3%). The mean incidence of clinical signs, mortality, and lethality rates were 8.9%, 1.7%, and 23.0%, respectively. Interestingly, mortality and lethality were significantly higher in dairy cattle than in beef (OR of 3.0 and 4.4, respectively). Oral lesions, dysphagia, and hoof problems were common clinical signs found in the study. Among all, the presence of tongue edema, dehydration, and dyspnea was associated with higher lethality rates. These results highlight a significant impact of EHD during the emergency. In fact, it must be noted that our data are based on passive surveillance, so true incidences are likely to be higher. The clinical signs and epidemiologic characteristics reported in this paper may contribute to understanding the clinical implications of the disease in cattle and help anticipate the effects of EHD virus (EHDV) introduction into naïve regions.

## 1. Introduction

Epizootic hemorrhagic disease (EHD) is an infectious, noncontagious viral disease of wild and domestic ruminants caused by the EHD virus (EHDV). Currently, EHDV is spreading in Europe, having been introduced for the first time in 2022. EHDV is an *Orbivirus* belonging to the *Sedoreoviridae* family, closely related to the bluetongue virus (BTV), African horse sickness virus, and equine encephalosis virus [1] and transmitted by biting midges of the genus *Culicoides*. Up to date, the virus has seven recognized serotypes (1–2 and 4–8) [2], although it is likely that additional serotypes exist.

The distribution of EHDV is constrained by the range of competent vectors, yet it has been detected across both tropical and temperate regions on all continents (approximately between latitudes 35° S and 49° N) [3]. Prior to 2022, however, no EHD cases had been reported in Europe. In October 2022, the first cases on the continent were reported in Italy (Sicily and Sardinia) and shortly thereafter, in November 2022, in Spain [4], affecting both cattle and other domestic and wild ruminants [5]. In Spain, EHDV-8 continued to spread to northern Spain during the subsequent vector season in 2023, reaching Portugal and southwestern France. The origin of the emergency is believed to be related to the emergence of a new strain in Tunisia in 2021 (EHDV-8/17 TUN2021) [6], which was later identified in the outbreak in Italy [7]. To date, EHDV-8 has been the only serotype identified in outbreaks in Europe [4].

EHD has traditionally been associated with wild cervids, particularly white-tailed deer (*Odocoileus virginianus*) in North America, where EHDV has become enzootic and causes periodic outbreaks with high morbidity and mortality rates [8]. Cattle are reservoir hosts for the virus [9] and were traditionally believed to be less susceptible to clinical signs [3]. In some endemic regions, clinical disease in cattle is reported to be uncommon, although it can become significant on a regional basis when an outbreak occurs [10]. Thus, several important outbreaks have been recurrently reported in North America [10, 11] or Japan [12–14] among other countries. However, the clinical significance of EHD in cattle is believed to have increased in the last decades. Thereby, multiple outbreaks involving serotypes 1, 6, or 7 have been documented in diverse countries such as Algeria, Tunisia, Morocco, Israel, Jordan, or Turkey [15, 16]. Moreover, these outbreaks have become more frequent, virulent, and widespread since the range of distribution of EHDV has expanded and reached new areas [3].

Clinically affected cattle are typically described as having fever, anorexia, facial edema, dysphagia, ulcerative and necrotic lesions of the mouth, reduced rumination, respiratory distress, hyperemia of teats, and udder, difficulty swallowing, lameness, a drop in milk production, and reproductive disorders [3, 9]. The disease may also have a significant economic impact. The most comprehensive analysis of EHD-associated losses has been conducted during the EHDV-7 outbreak in Israel in 2006, which estimated losses of US $26.5/cow due to milk loss and involuntary culling [17]. Recent studies also reported significant production losses in cows with moderate-to-severe affectation [18]. However, the impact of EHD on cattle farms in Spain has not been comprehensively detailed. Therefore, in this study, we aim to evaluate the incidence of clinical disease in cattle farms in northwestern Spain and describe the clinical signs and their frequency during the early stages of the emergency. This may contribute to a better understanding of the clinical implications of the disease in cattle and help anticipate the effects of EHDV introduction into naïve regions.

## 2. Material and Methods

### 2.1. Description of the Area of Study and the Emergence of EHD

The study was conducted in Galicia (northwestern Spain). This region plays a significant role in Spain's overall cattle production, with 32% of cattle farms, 19% of the total cattle population, and ~41% of the dairy cattle in Spain [19]. Most farms in Galicia are small-scale, with 49% housing fewer than 10 animals, meaning that a minority of farms concentrate the bulk of production. Despite its prominence in the Spanish dairy industry, Galicia also has a substantial beef cattle sector. In fact, among farms with more than 10 animals, 58% are beef-oriented.

There are differences in husbandry practices and breeds between dairy and beef production. Dairy farming is predominantly intensive, with Holstein-Friesian being the main breed, whereas beef cattle are raised in semi-extensive systems with continuous grazing, and breeds are mainly *Rubia Galega*.

**Figure 1:**
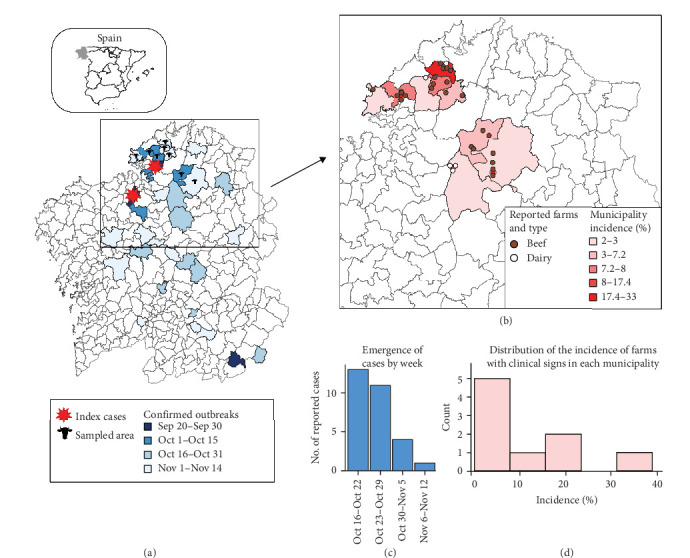
(a) Spatial distribution and temporality of EHD confirmed cases in NW Spain by the end of 2023; (b) spatial distribution of the sampled farms (*n*=29) and incidence of clinical signs by municipality; (c) temporality of the beginning of cases in the sampled farms; (d) distribution of the municipality-level incidence of clinical signs.

EHD was first detected in Galicia on September 9, 2023 [5]. Since then, EHDV rapidly spread throughout the region, causing confirmed cases across Galicia within 2 months (Figure 1). The last outbreak in 2023 was declared on November 14, although the disease re-emerged in the region throughout 2024.

### 2.2. Data Collection

We conducted this study to investigate the clinical outcomes in affected areas. To do so, in December 2023, we reached out to veterinarian practitioners across the northwestern region where the initial EHD outbreak had emerged and asked them to report which farms under their care had officially confirmed EHDV cases. Since EHD is a notifiable disease in the European Union [20], suspected cases had to be reported to the regional authorities. Once notified, the authorities collected blood samples from symptomatic animals within the herd and tested them for EHDV using PCR to confirm the presence of the virus. In this process, not all animals on a farm were necessarily tested. Therefore, we defined a farm as EHD positive when the presence of EHDV was officially confirmed in at least one animal in the herd following this process, and these were the farms included in this study.

We designed an epidemiological survey to gather information on the demographics and characteristics of these farms and to describe the clinical signs and outcomes found in positive farms (Supporting Information 1). We registered the clinical signs found on the farm and quantified their frequency in each farm using a six-level scale: no present, present in 2–5 animals, in less than a third, about half, about two-thirds, all animals. Abortion was defined as the death and expulsion of a fetus observed by the farm and was recorded during the outbreak period (September–December 2023). We also recorded which age groups exhibited clinical signs on each farm (calves, heifers, cows with one to four parturitions, cows with more than four parturitions). Additionally, we defined a variable to categorize the severity and duration of the cases into four classes: (i) no mortality and short-duration cases (<10 days): NM-SD; (ii) mortality and short-duration cases: M-SD; (iii) high mortality (>2%) and cases appearing during >10 days: HM-LD; and (iv), low or no mortality but prolonged clinical signs in affected animals: NLM-LD. We assigned each farm to one of these categories when the majority of affected animals (>75%) fell within a single category. We also collected data on the type of production (beef or dairy cattle), grazing practices (continuous or intermittent [i.e., keeping animals in pens during some parts of the day]), and the presence/absence of other ruminants on the farm. Furthermore, we calculated farm-level epidemiological parameters: farm size, onset of clinical signs, incidence of clinical signs, mortality, and lethality rates.

In addition, we retrieved population data from official sources regarding the number of farms per municipality [21] to calculate the regional incidence of affected farms and information on the municipalities with confirmed outbreaks [5].

Although participation in the study was voluntary, no farm refused to share data, but we limited the farms included in the study to those with more than 10 animals. Smaller farms were excluded because, in this design, it is harder for us to determine whether they were infected, as we relied on veterinarians to report confirmed cases, and these farms may be less likely to seek veterinary consultation and monitoring.

### 2.3. Statistical Analysis

The farm epidemiological parameters were calculated as follows: the incidence of clinical signs as the percentage of animals with clinical signs out of the total census; mortality rate as the percentage of dead animals of the total census; and lethality rate as the percentage of dead animals of the animals with clinical signs. These values were calculated from the start of the outbreak on the farm (moment of detection of the first animal with clinical signs as perceived by the farmer) until the moment when the survey was filled up (December 2023). To obtain the incidence of farms with clinical signs in each municipality, we calculated the percentage of positive farms by the number of farms with >10 animals in each municipality.

We ran mixed logistic regression models to analyze factors influencing the incidence of clinical signs, and mortality and lethality rates. The dependent variables (incidence, mortality, and lethality rates) were binomial: clinical cases/census, dead animals/census, and dead animals/animals with clinical signs, respectively. They were analyzed in three logistic mixed regression models (one for each dependent variable) where the explored fixed effects were factors: farm type (dairy vs. beef), grazing system (continuous grazing vs. intermittent grazing), and presence of other ruminants (yes vs. no); and the random factor was the municipality where the farms are located.

We also analyzed the relationship of lethality rates (dependent variable) with the presence or not of animals of each clinical sign in univariate mixed logistic regression models. We ran separate univariate models for each clinical sign, where the presence/absence of each sign was the fixed factor, and the municipality was used as a random factor. The analysis was performed using the package glmmTMB [22] in the statistical software R v.4.3.0 [23].

## 3. Results

We received information from 29 farms with >10 animals. Clinical signs were first detected on a farm on October 15th, and the last reported case occurred on November 6th. The estimated incidence of farms with clinical signs was 6.2% for the entire study area, but it reached as high as 33.3% in some municipalities (Figure 1). Most confirmed cases occurred in beef farms (*n*=24 82.8%). All reported farms practiced grazing; however, continuous grazing was exclusively observed in beef cattle. Since grazing type coincided with farm type classification, the variable grazing was not further considered.

Farm-level epidemiological parameters are shown in Table 1. We observed that the incidence of clinical signs in each farm was generally low (median = 6.7). In beef farms, incidence values seemed to be higher, but we did not find statistically significant differences between the two types of farms (OR = 1.0; 95% CI = 0.6–1.7; *p*=0.952). Conversely, dairy farms exhibited significantly higher mortality (OR = 3.0; 95% CI = 1.2–8.0; *p*=0.023) and lethality (OR = 4.4; 95% CI = 1.7–11.6; *p*=0.002) (variance of random effects in the models were 0.05, <0.01, and 0.16, respectively). No statistical relationship was found between the presence of other ruminant species on farms and any epidemiological parameters considered.

**Figure 2:**
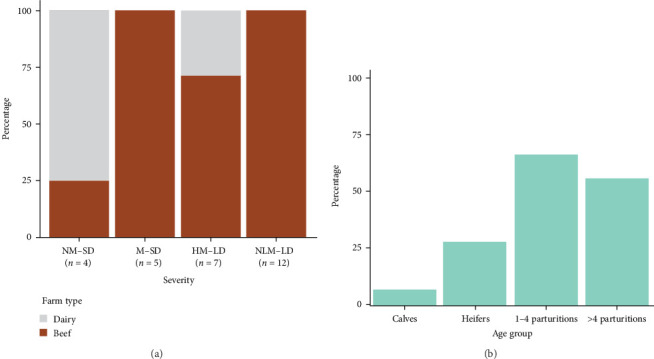
(a) Percentage of farms that presented clinical signs according to their farm type and severity: NM-SD no mortality and short-duration; M-SD: mortality and short-duration; HM-LD: high mortality and long duration; NLM-LD: no or low mortality and long duration; (b) percentage of farms that presented animals with clinical signs in each age group.

**Figure 3:**
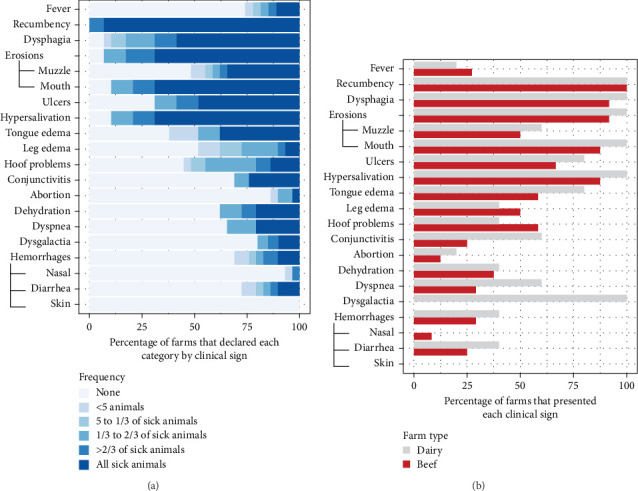
(a) Percentage of farms that declared each clinical sign in each category of frequency; (b) percentage of farms that presented each clinical sign by farm type.

**Figure 4:**
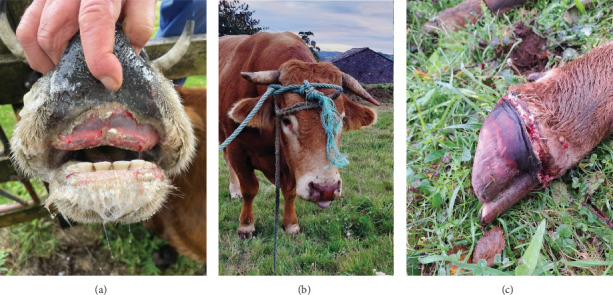
(a) Animal showing erosions and ulcers in mouth along with hypersalivation; (b) reddening and ulcers in the muzzle, tongue edema with tongue protruded; (c) coronary band injury and shedding of the hoof capsule.

Infection on farms was generally mild, that is, no mortality and short-duration cases. Cases with mortality were more common in dairy farms, especially with short duration (Figure 2a). Episodes of significant mortality occurred, when they occurred, tended to be more persistent in beef farms (Figure 2a). Regarding age, clinical signs were more frequently observed in adults (Figure 2b). The reported clinical signs and their frequencies are shown in Figure 3. Apart from recumbency, the most frequent clinical signs were associated with oral cavity lesions. Oral erosions were also found, primarily in the mouth (89.7% of farms), along with hypersalivation (89.7%) and tongue edema (62.1%) (Figure 4a,b). Oral lesions often progressed to ulcers (69.0% of farms). Dysphagia was present on nearly all farms and on most of the affected animals.

About half of the farms also reported leg edema (48.3%) and hoof problems (55.2%) (Figure 4c). However, when present, these issues appeared to be less widespread across the farm compared to other clinical signs). Hemorrhagic lesions were also detected but were less common and mainly related to the presence of blood in the feces (27.6%). Other conditions such as dyspnea, conjunctivitis, dehydration, and abortion presented lower percentages, while dysgalactia was detected in all dairy herds. Interestingly, when comparing the incidence of these problems between farm types, most clinical signs were more frequently reported in dairy farms, except for leg edema and hoof problems, which were more common in beef farms (Figure 3b).

When we studied the relationship between lethality rates and the presence of specific clinical signs on a farm, we found higher lethality rates when tongue edema (OR = 8.6), dyspnea (OR = 3.4), or dehydration (OR = 3.0) were present on the farm (Table 2). No statistical associations were found with the other clinical signs.

## 4. Discussion

In this paper, we report the clinical outcome of EHD on cattle farms in NW Spain during the fall 2023 emergence. This study is valuable to provide an initial overview of the clinical impact of the introduction of this virus into Europe. Our results indicate a significant occurrence of EHD-related clinical signs in the region and suggest that cattle were highly exposed to the virus during the outbreak. In fact, in three out of nine municipalities, more than 15% of the farms with >10 animals exhibited clinical signs (Figure 1). Moreover, it is important to note that our data were based on passive reporting of confirmed cases by veterinarians. It is possible that some farms with very mild cases may not have noticed and reported the infection, meaning the true incidence is likely higher. This highlights the substantial impact of the EHD emergency and aligns with the rapid and widespread dissemination of the virus across the country [24]. A similar situation has been later observed in other regions, such as France, where by the end of 2024, more than 4000 outbreaks had been reported since the emergence in September 2023 [25].

Epidemiological indicators from EHD outbreaks are scarce in the literature. However, the available data from past decades show a wide range of morbidity rates (e.g., 5%–80% in Yadin et al. [26]; 1%–12% in Garret et al. [10]; or 0%–13% in Omori et al. [12]), highlighting the variability of EHD's impact on farms. Similarly, we predominantly found a low within-herd incidence rate of clinical animals (mean = 9.4%). Our mortality rates were slightly higher than these studies. For example, 0.2% and 1% of mortality in [12] and in [10]. Kedmi et al. [17] found an average excess mortality and involuntary culling of 1.47/100 cows. Case-fatality rates were also lower in [12]; 10.3%. Therefore, the course of EHD in the observed farms was similar or slightly worse in the average farm of our study (Table 1). However, we found that the severity and duration of cases varied among farms. Thus, most of the affected farms had animals with mild signs that lasted only a few days, consistent with previous observations [3]. However, a notable number of farms experienced more severe cases (16/29; Figure 2a), ranging from a prolonged convalescence to acute courses, with different degrees of mortality. The factors influencing those different courses could not be determined, but we found that higher mortality and lethality rates were observed in dairy cattle. This was noteworthy, as we would expect their exposure to vectors to be lower compared to beef cattle since dairy farms typically house animals, which is suggested to reduce the risk of *Culicoides* spp. bites [27]. Given all these considerations, this finding may point to breed-specific differences in viral pathogenicity; nevertheless, the number of dairy farms in our study was low, and we were unable to collect animal-level data, so other potential predictors may have gone undetected. Factors such as the lactation stage at the time of infection, production level, or immunosuppression may also influence infection outcomes, but these could not be assessed in our study. In this regard, it is important to note that grazing practices were strongly correlated with farm type, making it difficult to disentangle whether the observed effects were due to farm type-related factors or grazing itself. Additional studies are required to clarify whether breed-specific susceptibility exists, but our findings suggest that this possibility should be explored. Strain may also contribute to pathogenic variation. EHDV has demonstrated the ability to reassort, allowing it to adapt to different environmental conditions and potentially lead to unpredictable disease features [3]. These reassortment events have even been linked with the emergence of some outbreaks [3]. However, only EHDV-8 has been detected in the study area so far. The circulating sequence has been clustered with sequences from different serotypes that have been circulating in Africa for a long time. More research is needed, but current data suggest that reassortment of local African strains may have driven this emergency [28].

Calves and young animals were less likely to exhibit clinical signs. This may be due to a lower susceptibility to the virus or environmental factors, including management practices (e.g., more housing time for young animals) and vector preferences. A similar age-related pattern has been observed in past emerging diseases transmitted by *Culicoides* spp., such as Schmallenberg's disease, and was hypothesized to be influenced by host preferences of the midges [29]. For example, attractiveness to the vector depends on host size [30], and larger body-sized adults can also emit larger quantities of some chemical attractants, such as carbon dioxide or other volatile chemicals [31], although the most effective attractants vary depending on the *Culicoides* species [32]. However, further research is needed to determine whether the underlying cause is pathophysiological or environmental.

The pathogenic course of the infection is not well known since experimental challenges with different serotypes had not been very successful in reproducing the clinical signs observed in the field, regardless of the breed. However, Spedicato et al. [33] inoculated five Holstein-Friesian calves with EHDV-8 from the Europe emergency, resulting in fever in three out of five and ulcerative lesions of the muzzle in one. It is expected that EHDV will initially replicate in the lymph tissue local to the entry site, phagocytes, and in the endothelial cells. This results in vascular injury and, hence, edema, hemorrhage, tissue necrosis, and thrombocytopenia [34].

Clinical signs were consistent with those previously reported, for example, in outbreaks in Japan [12], the Mediterranean Basin [6, 15, 26] and North America [10]. Thus, fever, oral ulceration, excessive salivation, lethargy, recumbency, hyporexia, reduced milk production, lameness, and edema were frequently found here in agreement with previous descriptions. Among these, oral and hoof problems are traditionally viewed as the more predominant clinical outcome [9], which was also the case in our study. Dysphagia was frequently observed in our study and may result from erosive lesions or tongue edema. Besides, it can stem from lesions in the striated muscles of the esophagus, larynx, pharynx, or tongue [12]. Animals with a protruded, swollen, tiff tongue exhibit a more marked deglutitive difficulty and an impaired ability to drink and eat properly [12]. In severe cases, this leads to dehydration and anorexia (Supporting Information 2), which may explain the association between higher farm lethality rates and outbreaks in which tongue edema and dehydration occurred. We also found significantly higher lethality rates when animals presented dyspnea. EHDV can rapidly spread to other tissues from the entry sites, in particular to the lungs and spleen [35]. The development of a respiratory problem may exacerbate the disease course. Lung infection can result in pulmonary edema, contributing to respiratory distress and secondary pneumonia. Moreover, since EHDV infects lymphocytes, it may induce temporary immunosuppression, potentially increasing susceptibility to other respiratory diseases.

Hoof problems are also a common manifestation of EHD [9]. The virus is reported to cause lameness due to corionitis, which can result in recumbency and anorexia. In our study, the presence of hoof problems did not correlate with more severe courses at the farm level, nor were they associated with higher farm-level lethality rates. However, varying degrees of hoof problems were observed, including prolonged severe cases that led to death or culling, but we cannot infer the farm-level impact of these severe cases due to our farm-level design and the limited number of such cases. Other farms with outbreaks in other regions showed erosive lesions in the udder or in the vulva [6, 36], but we did not detect them, so they might be less frequent or have a presentation influenced by other factors.

In terms of productivity effects associated with EHD, milk loss, and involuntary culling have been pointed out as significant consequences of the disease [17]. In our study, dairy farms also complained about dysgalactia and a decrease in milk yield of about 10%–20% in affected animals (data not shown). Milk losses of 1.73–4.76 kg have also been reported in Spain [18, 36]. Recio et al. [18] also reported that cows with severe clinical signs had a significantly higher risk of being culled, in agreement with Kedmi et al. [17]. Reproductive loss may also be a consequence of EHDV infection. Although we did not analyze individual animals, the percentage of farms that reported abortion problems was low (Figure 3), suggesting that the overall incidence of abortion due to EHDV infection is expected to be low compared to other potential outcomes of the disease.

Given the predominance of lesions on the mouth and hoof, these sites should be primarily examined when looking for the presence of EHD, and this disease should be included in the differential diagnosis of Bovine viral diarrhea or bluetongue in Europe. The latter is transmitted by the same vector and has also re-emerged in the region lately [37]. Thereby, it would be interesting to evaluate whether clinical significance could worsen if Bluetongue and EHD are concurrent in the animal. However, by the time of the study, BTV was not present in the study area, though it emerged soon afterward [37]. The prospectives for the disease are uncertain. If a significant number of animals have already been exposed, the clinical impact in the next flying-season for vectors is expected to decrease. However, the fact that wild ruminants may also get infected with the virus may favor the establishment of an endemic situation, as it occurs in other countries in similar latitudes, such as the United States, where EHDV persists in wild ruminant reservoirs and leads to periodic outbreaks [8, 10]. In those scenarios, the risk of cattle developing clinical disease in the future depends on the infection pressure, which results from the combination of *Culicoides* spp. abundance, viral persistence in wild ruminant populations, and favorable climatic conditions (temperature, rainfall, and humidity) [38]. The progressive expansion of the range of activity of vectors in the last decade also poses a risk for recurrent emergences and for the introduction of other EHDV serotypes that are present in Northern Africa (e.g., EHDV-6 in Morocco [3]). They may still cause disease problems since there is apparently no good cross-protection between them [3, 39]. Thus, vaccination, vector-oriented preventive measures, and early identification of infection are crucial measures to minimize losses due to EHD.

## 5. Conclusion

Our results suggest that the infection with EHDV is widespread in the region despite the recent introduction. Moreover, EHDV has caused a significant clinical impact on some farms, even resulting in high mortality and lethality rates in some cases. These results highlight a significant impact of EHD when it was introduced to the naïve populations of NW Spain. The clinical signs and epidemiologic characteristics reported in this paper may contribute to understanding the clinical implications of the disease in cattle and help anticipate the effects of EHDV introduction into naïve regions.

## Figures and Tables

**Table 1:** Census, farm-level incidence of clinical signs, mortality, and lethality rates by farm type in cattle from NW Spain (*n*=29).

Farm type	Animal census (*n*)		Cases by farm (*n*)		Incidence (%)		Mortality (%)		Lethality (%)	
	Mean	Median (range)	Mean	Median (range)	Mean	Median (range)	Mean	Median (range)	Mean	Median (range)
Beef	36.5	30 (10–93)	3.0	2 (1–11)	9.4	6.7 (2.5–33.3)	1.3	0 (0−12.0)	15.7	0 (0–100)
Dairy	68.8	54 (40–140)	5.6	3 (1–18)	6.5	5 (2.0–12.9)	3.4*⁣*^*∗*^	2.5*⁣*^*∗*^ (1.7–7.1)	57.8*⁣*^*∗*^	50*⁣*^*∗*^ (33.3–100)
Total	42.1	40 (10–140)	3.5	2	8.9	6.7 (2.0–33.3)	1.7	0 (0–12.0)	23.0	0 (0–100)

*⁣* ^*∗*^Statistical differences between farm type (*p* < 0.05).

**Table 2:** Results of the mixed logistic regressions between lethality rate and the presence or not of specific clinical signs in a farm in which a significant association was found (*P* < 0.05).

Clinical sign	No. of farms	*p*-Value	OR	95% CI
Tongue edema	18	0.006	8.6	1.8–40.6
Dehydration	11	0.035	3.0	1.1–8.5
Dyspnea	10	0.043	3.4	1.2–9.9

## Data Availability

The data that support the findings of this study are available from the corresponding author upon reasonable request.

## Supporting Information

Supporting Information 1 Epidemiological survey

Supporting Information 2 Cow showing difficulty to drink due to tongue protrusion
