# Supplementary material for: Description of the Clinical Findings Associated With the Epizootic Hemorrhagic Disease in Cattle From Northwestern Spain During the Emergence
Source: Transbound Emerg Dis. 2025 May 24;2025:7808243. doi: 10.1155/tbed/7808243 (PMC12126260; doi:10.1155/tbed/7808243)
Supplement: Supporting Information 1 — Epidemiological survey [file 7808243.f1.pdf]

Survey on Clinical Signs of Epizootic Hemorrhagic Disease

Section 1

General Description of the Farm

1. Name of the person completing the survey

   ____

2. Name/identification of the farm

   ____

3. Municipality of the farm: ____
4. Type of farm: dairy/beef: ____
5. Farming system: intensive/extensive/semi-extensive: __________________
6. Outdoor access: permanent housing/pasture access/other outdoor outings:

   ____

7. Do the animals go outdoors?

   ☐ No, permanent housing

   ☐ They have pasture access

   ☐ The animals are always in the pasture

9. Which animals go outdoors? Specify if adult cows/dry cows/heifers or all animals: ____
10. Number of cattle: _______________________
11. Breed of the animals on the farm?: ________________________
12. Are sheep/goats also present on the farm? Yes/No _____________________
13. Is there a confirmed case of EHD? Yes/No: ____________________________

Section 2

Onset of Clinical Signs

14. When did the signs first appear? (Date)

____

15. Date of sampling of the first confirmed animal

____

16. Number of affected animals. Indicate the number of animals with confirmation of EHDV and number of animals with clinical signs observed since the emergence of EHD

    - Confirmed: _______________
    - Clinical signs: _____________

      If known, fill the following:

      - Number of animals with mild clinical signs:

        Mild: fever, light nasal or conjuctival excretions

      - Number of animals with severe clinical signs:

        Of this, how many?

        - Recovered __________
        - Sequealae ___________

17. Number of deceased animals: ___________

18. At what ages have you observed clinical signs?

    ☐ Calves

    ☐ Heifers

    ☐ Animals after their first calving

    ☐ Animals after their fourth calving

Section 3

Observed Clinical Signs

How many animals have been observed with the following clinical signs? If availabe fill with the actual number of animals with each condition (in n column), if don't, mark the correspondent cell using the following code: A = None / B= fewer than 2 animals / C = less than one-third of the animals / D = between one-third and two-thirds / E = more than two-thirds /F = all

	n		A		B		C		D		E		F	
	m	s	m	s	m	s	m	s	m	s	m	s	m	s
Difficulty drinking normally														
Mouth erosions or ulcers														
Muzzle erosions or ulcers														
Mouth ulcers														
Muzzle ulcers														
Fever														
Tongue protruding from the mouth														
Excessive salivation														
Swollen legs														
Hoof lesions														
Conjunctivitis or eye lesions														
Abortions*														
Dehydration														
Respiratory difficulty														
Pneumonia														
Decreased milk production														
Nasal hemorrhages														
Bloody diarrhea														
Skin hematomas														
Udder ulcers														

Other clinical signs you wish to report

____

____

____

____

How many affected animals recovered milk production in less than two weeks?

____

Section 4

Course of the infection

19. On average, how long do the signs last?

    ☐ < 10 days

    ☐ > 10 days

    ☐ > 20 days

20. How many animals show mild signs and recover in < 10 days? ______

21. How many animals show clinical signs and die in < 10 days? _______

22. How many animals show clinical signs lasting > 10 days didn't die? _____

23. How many animals show clinical signs lasting > 10 days and died? _______

If some animals presented an unusual or very different course from above, please fill:

Animal	Duration of signs	Recovered /sequalae/ death?	Clinical signs observed

Section 4

Other Diseases

24. Is this farm enroled in a Health Defense Association? ____________________

25. Are any of the following present?

    - ☐ BVD
    - ☐ Paratuberculosis
    - ☐ IBR
    - ☐ Bluetongue
